# Quick Epidural Top-up with Alkalinized Lidocaine for emergent caesarean delivery (QETAL study): protocol for a randomized, controlled, bicentric trial

**DOI:** 10.1186/s13063-023-07366-1

**Published:** 2023-05-19

**Authors:** Thomas Lechat, Thomas d’Aprigny, Jérémy Henriot, Jill Arthur, Dienabou Sylla, Antoine Bénard, Karine Nouette-Gaulain

**Affiliations:** 1 Department of Gynecological and Obstetrical Anesthesiology, Bordeaux University Hospital, Bordeaux, France; 2 Department of Anesthesiology, Bordeaux University Hospital, Bordeaux, France; 3 Department of Anesthesiology, Centre Hospitalier de la Côte Basque, Bayonne, France; 4 Department of Clinical Epidemiology, Bordeaux University Hospital, Bordeaux, France

**Keywords:** Epidural, Sodium bicarbonate, Insufficient analgesia, Local anesthetic, Emergency caesarean section, Labor, Lidocaine

## Abstract

**Background:**

General anesthesia in pregnant women can be associated with significant maternal and fetal morbidity. Emergency caesarean section can be performed by converting labor epidural analgesia to surgical anesthesia by injecting high-dose short-acting local anesthetics through the epidural catheter. The effectiveness and the delay to obtain surgical anesthesia depends upon the protocol used. Data indicate that alkalinization of local anesthetics may shorten their onset of action and increase their effectiveness. This study investigates whether alkalinization of adrenalized lidocaine could increase the efficacy and decrease the delay of onset of surgical anesthesia via an indwelling epidural catheter, thus decreasing the necessity to resort to general anesthesia for emergency caesarean deliveries.

**Methods:**

This study will be a bicentric, double-blind, randomized, controlled trial with two parallel groups of 66 women who require emergency caesarian deliveries and who have been receiving epidural labor analgesia. The number of subjects in groups will be unbalanced with a 2:1 ratio of experimental:control. In both groups, all eligible patients will have had an epidural catheter placed for labor analgesia with levobupicaine or ropivacaine. Patient randomization will occur when the decision is made by the surgeon that an emergency caesarean delivery is indicated. Surgical anesthesia will be obtained by injecting 20 mL of 2% lidocaine with epinephrine 1:200,000, or 10 mL 2% lidocaine with epinephrine 1:200,000 plus 2 mL sodium bicarbonate 4.2% (total of 12 mL). The primary outcome will be the rate of conversion to general anesthesia for failure of the epidural to provide adequate analgesia. This study will be powered to detect a 50% reduction in the incidence of general anesthesia, from 80 to 40%, with a confidence ratio of 90%.

**Discussion:**

Sodium bicarbonate could be used to avoid general anesthesia for emergency caesarean deliveries by providing reliable and effective surgical anesthesia in women with pre-existing labor epidural catheters is promising. This randomized controlled trial seeks to determine the optimal local anesthetic mixture for converting epidural analgesia to surgical anesthesia for emergency caesarean sections. This may decrease the need for general anesthesia for emergency caesarian section, shorten the time to fetal extraction, and improve safety and patient satisfaction.

**Trial registration:**

ClinicalTrials.gov NCT05313256. Registered on 6 April 2022

**Supplementary Information:**

The online version contains supplementary material available at 10.1186/s13063-023-07366-1.

## Administrative information

Note: the numbers in curly brackets in this protocol refer to SPIRIT checklist item numbers. The order of the items has been modified to group similar items (see http://www.equator-network.org/reporting-guidelines/spirit-2013-statement-defining-standard-protocol-items-for-clinical-trials/*).*

**Table:** 

Title {1}	Quick Epidural Top-up with Alkalinized Lidocaine for Emergent Caesarean Delivery (QETAL study): protocol for a randomized, controlled, bicentric trial
Trial registration {2a and 2b}.	ClinicalTrials.gov NCT05313256 (April 6, 2022)
Protocol version {3}	Version 1.2 (March 15, 2022)
Funding {4}	Full financial support from the University Hospital of Bordeaux.
Author details {5a}	Thomas Lechat, MD, Department of Gynecological and Obstetrical Anesthesia, Bordeaux Thomas d'Aprigny, MD, Resident in Anesthesiology, Bordeaux Jérémy Henriot, MD, Department of Anesthesiology, Bayonne Jill F. Arthur, MD, Department of Anesthesiology, Bordeaux Dienabou Sylla, MSc, Department of Clinical Epidemiology, Bordeaux Antoine Bénard, MD, PhD, Department of Clinical Epidemiology, Bordeaux Karine Nouette-Gaulain, MD, PhD, Department of Gynecological and Obstetrical Anesthesia, Bordeaux
Name and contact information for the trial sponsor {5b}	Bordeaux University Hospital, Place Amélie Raba Léon, 33076 Bordeaux, France
Role of sponsor {5c}	The funding body was not involved in the study design, collection, analysis, interpretation of data, the writing of this article, or the decision to submit it for publication.

## Introduction

### Background and rationale {6a}

Epidural analgesia is performed throughout the world for intrapartum pain relief. Approximately 85% of women in France use epidurals during labor [1]. In the case of emergency caesarean section, surgical anesthesia can be obtained by injection of a rapid-onset local anesthetic (LA) via an existing epidural catheter. If insufficient analgesia is obtained by this method, the patient must receive a general anesthetic. General anesthesia for emergency caesarean delivery is associated with significant maternal and fetal morbidity, including a higher incidence of difficult oro-tracheal intubation [2, 3], aspiration of gastric contents [4], and neonatal respiratory depression [5].

The French Society of Anesthesia and Intensive Care Medicine (Société Française d’Anesthésie et de Réanimation, SFAR) [6] provides guidelines for the use of an epidural catheter that has been placed for labor analgesia to convert to surgical anesthesia (“epidural conversion”) for emergency caesarean deliveries with the addition of 15 to 20 ml of 2% lidocaine plus adrenaline 1:200,000 to an existing epidural. The onset of surgical anesthesia is usually seen within 10 to 15 min. In cases of insufficient analgesia, general anesthesia will be required. Therefore, the key to avoiding a general anesthetic in this population is the rapid and predictable onset of surgical anesthesia via an indwelling epidural catheter.

The addition of sodium bicarbonate to commercially available lidocaine increases its pH towards the physiological range. Theoretically, this would enhance the speed of onset of a lidocaine nerve block, but this effect remains controversial in animal models and in clinical practice. In preparations of rat sciatic nerve, the effect of lidocaine was enhanced in the presence of a carbon dioxide-rich microenvironment [7]. In clinical practice, however, the addition of sodium bicarbonate to lidocaine is used in 35% of labor epidural conversions to surgical anesthesia in Denmark [8] and in 12% of epidural conversions in the UK [9]. Although clinical studies have shown the efficacy of this technique in scheduled caesarean sections [10–12], only two randomized, controlled trials have been conducted specifically in the context of emergency caesarean delivery during labor. In the first [13], the addition of 1.2 ml 8.4% sodium bicarbonate to a mixture of 15 ml 2% lidocaine with epinephrine 1:200,000 plus fentanyl 75 μg decreased the delay required to obtain a T6 epidural anesthesia by 50% (5.2 min vs. 9.7 min [*p* < 0.001]), without maternal or fetal adverse effects. In the second study [14], a mixture of 1.8% lidocaine with epinephrine 1:200,000 plus 0.76% bicarbonate was compared to levobupivacaine. The onset of T5 block (bilateral sensory block to touch at the T5 dermatome level) was shorter in the epidural mixture group. More recently, Reschke et al.’s 2019 meta-analysis examined the six most commonly used epidural extension solutions across 1280 patients in 24 randomized controlled trials. Using a network Bayesian analysis of time to epidural anesthesia, the authors found lidocaine 2% alkalinized with bicarbonate as the top-ranking solution for speed of epidural anesthesia [15].

The main hypothesis to explain this pharmacokinetic effect is that lidocaine molecules exist in a greater proportion of its non-ionized form in an alkaline environment. The non-ionized form is the only one capable of crossing the perineural membrane and inducing a conduction block of sodium channels [16].

For emergency caesarean delivery, there are many combinations that can be used with 2% lidocaine. In our unit, we mainly use two combinations, according to the choice of the practitioner: 20 ml of 2% lidocaine with epinephrine 1:200,000 or a 12 mL mixture of lidocaine plus bicarbonate (10 mL of 2% lidocaine with epinephrine 1:200,000 plus 2 mL 4.2% sodium bicarbonate ). This last combination was adopted as a result of previous preliminary studies that showed rapidly established surgical anesthesia. However, the incidence of failure to achieve surgical anesthesia with a need for conversion to a general anesthetic for emergency caesarean section has not been described. We conducted a preliminary audit of our clinical practice between January 2019 et July 2019 where 51 patient files were randomly drawn and analyzed. The evaluation was conducted from a prospectively collected database of patient paper records with data collection completed from the electronic medical record software used at the CHU of Bordeaux, Dxcare® (Medasys) [17]. When sodium bicarbonate was added to the mixture through the epidural catheter, there was a decrease of 50% in the time to surgical anesthesia (5 min vs 10 min [*p* < 0.001]), as published in previous studies [13, 14]. Although the sample size is small, in emergency caesarean delivery (fetal extraction in under 15 min), the use of bicarbonate to convert epidural analgesia to surgical anesthesia was associated with a decrease in the incidence of general anesthesia by 80% (4/4 vs 0/7 [*p* = 0.003]).

### Objectives {7}

Based on the results of the preliminary audit, we plan to conduct the QETAL study. This bicentric, randomized controlled patient-blinded clinical trial will focus on parturients who have an epidural catheter in place for labor analgesia and require emergent fetal extraction (<15 min). Our hypothesis is that adding sodium bicarbonate to the lidocaine-epinephrine solution administered through the epidural catheter will reduce the rate of conversion to general anesthesia due to faster onset of surgical epidural anesthesia.

General anesthesia in this context is defined as intravenous induction of general anesthesia and tracheal intubation between the attempted conversion of epidural analgesia to surgical anesthesia via the epidural catheter and umbilical cord clamping. The primary objective is to evaluate whether alkalinization of lidocaine decreases the need for conversion to general anesthesia. Secondary objectives include comparing the delay to maternal incision and fetal extraction, maternal and fetal safety criteria, the level of anesthesia assessed 1 h after surgical incision, and maternal satisfaction with analgesia and anesthesia between the two groups.

### Trial design {8}

This QETAL study is a bicentric patient-blinded, two parallel-arms randomized, controlled, clinical trial of superiority. Patients will be randomly assigned in a 2:1 ratio in group LEB (lidocaine-epinephrine-sodium bicarbonate) and group LE (lidocaine-epinephrine) respectively.

## Methods: participants, interventions, and outcomes

### Study setting {9}

The patients will all be recruited from two academic hospitals, the University Hospital of Bordeaux and the Bayonne Hospital, southwestern France.

### Eligibility criteria {10}

Parturients with the following criteria will be included: adult patients (age ≥ 18 years) who are affiliated with or beneficiary of French government-sponsored health insurance (sécurité sociale), having an initial indication for vaginal delivery, who require emergent fetal extraction via caesarean section (delay < 15 min), who are informed, and who have given their written consent according to the methods described in French Law [18], signed by the participant and the investigating physician at the latest after the therapeutic intervention.

Parturient with one or more of the following criteria will be not included: age less than 18 years, persons placed under guardianship, opposition to participation in the research before caesarean delivery, refusal or inability to consent, lack of understanding or significant language barrier, having another indication for general anesthesia defined by the following situations: non-functional epidural analgesia, consciousness disorders, eclampsia, suspected amniotic embolism, confirmed or suspected severe bleeding before birth, any contraindication to the use of the products defined in the protocol (adrenalized lidocaine; sodium bicarbonate).

### Who will take informed consent? {26a}

When eligibility to participate is validated, written information on the study is given during preoperative anesthesia consultation. In addition, a QRC code is given and video information can be consulted on YouTube (https://www.youtube.com/watch?v=JNabPb1vlRk). Upon the patient’s arrival in the hospital delivery room, the QRC code is displayed and the video can again be consulted. Before placement of the epidural catheter, the investigating physician verifies that the patient has understood the informational leaflet. In the case of emergency caesarean delivery (<15 min), the patient will have provided oral informed consent prior to the injection of either the LEB or LE mixture via the epidural catheter. After the caesarean delivery, informed written consent will be collected in the post-anesthesia care unit by the managing anesthesiologist.

### Additional consent provisions for collection and use of participant data and biological specimens {26b}

Not applicable. No biological samples and no additional consent provisions will be collected.

#### Interventions

##### Explanation for the choice of comparators {6b}

This study aims to compare the efficacy and onset time of epidural conversion between lidocaine-epinephrine (group LE) and lidocaine-epinephrine-sodium bicarbonate (group LEB) for emergency caesarean delivery.

In group LE, we will use 20 mL of 2% lidocaine with 1:200,000 epinephrine, which is the current recommendation of the SFAR and considered the gold standard in this circumstance [6].

In group LEB, we will add 2 mL of 4.2% bicarbonate to 10 mL of 2% lidocaine with 1:200,000 epinephrine. This specific mixture and volume have been used at Bordeaux University Hospital since 2016. While the volume of 12 mL may seem small compared to the usual volume used, we have observed in our unit that a volume greater than 12 mL of this solution often leads to nerve block higher than a T4 level. Despite lidocaine being a weak base (pKa=7.9), our preliminary study showed that the commercial preparation of 2% lidocaine with 1:200,000 epinephrine is acidic (pH 4). We conducted hourly measurements of the solution'’ pH over a 24-h period to confirm its stability and suitability for use (Table 1). Alkalinization of lidocaine can modify the non-ionized lidocaine and affect its potency. Moreover, the effectiveness of an injection into the epidural space is heavily influenced by the volume of the injected solution, unlike the intravenous administration route. Therefore, adding 8 mL of sodium chloride to the LEB group to equal the volume of the LE group (20 mL) could potentially result in a significant deviation from the desired level of spinal anesthesia achieved. Such a deviation would be inconsistent with our usual clinical practice, which involves using 12 mL to achieve a T4 level of anesthesia promptly.

**Table 1:** Measurements of pH were performed with a pHmeter (Mettler Toledo FiveEasy Plus^TM^ Series, Greifensee, Switzerland, Accuracy 0.01 pH unit) calibrated before each measurement with known solutions of pH 4 and 7

**Time (hour)**	**Solution**	**pH**
T (0)	2% lidocaine with 1:200,000 epinephrine	3.41
T (0)	4.2% *sodium bicarbonate*	7.84
T (0)	2% lidocaine with 1:200,000 epinephrine (10 mL) and 4.2% *sodium bicarbonate* (2 mL)	7.10
T (1)	2% lidocaine with 1:200,000 epinephrine (10 mL) and 4.2% *sodium bicarbonate* (2 mL)	7.10
T (2)	2% lidocaine with 1:200,000 epinephrine (10 mL) and 4.2% *sodium bicarbonate* (2 mL)	7.12
T (3)	2% lidocaine with 1:200,000 epinephrine (10 mL) and 4.2% *sodium bicarbonate* (2 mL)	7.12
T (4)	2% lidocaine with 1:200,000 epinephrine (10 mL) and 4.2% *sodium bicarbonate* (2 mL)	7.08
T (5)	2% lidocaine with 1:200,000 epinephrine (10 mL) and 4.2% *sodium bicarbonate* (2 mL)	7.14
T (6)	2% lidocaine with 1:200,000 epinephrine (10 mL) and 4.2% *sodium bicarbonate* (2 mL)	7.08
T (7)	2% lidocaine with 1:200,000 epinephrine (10 mL) and 4.2% *sodium bicarbonate* (2 mL)	7.05
T (8)	2% lidocaine with 1:200,000 epinephrine (10 mL) and 4.2% *sodium bicarbonate* (2 mL)	7.06
T (9)	2% lidocaine with 1:200,000 epinephrine (10 mL) and 4.2% *sodium bicarbonate* (2 mL)	7.06
T (10)	2% lidocaine with 1:200,000 epinephrine (10 mL) and 4.2% *sodium bicarbonate* (2 mL)	7.04
T (11)	2% lidocaine with 1:200,000 epinephrine (10 mL) and 4.2% *sodium bicarbonate* (2 mL)	7.06
T (12)	2% lidocaine with 1:200,000 epinephrine (10 mL) and 4.2% *sodium bicarbonate* (2 mL)	7.10
T (24)	2% lidocaine with 1:200,000 epinephrine (10 mL) and 4.2% *sodium bicarbonate* (2 mL)	7.08

##### Intervention description {11a}

When the decision is made by the obstetrician to perform an emergency caesarean section (delay <15 min), the anesthesiologist immediately proceeds to inject the epidural catheter with either:

- 20 ml of 2% lidocaine with epinephrine 1:200,000 or
- 12 ml of a mixture of 10 mL 2% lidocaine with epinephrine 1:200,000 plus 2 mL 4.2% sodium bicarbonate

##### Criteria for discontinuing or modifying allocated interventions {11b}

The experimental procedure in this clinical trial involves injecting a different type and volume of local anesthetic than the control group. This injection is given shortly after the participant’s study inclusion and randomization and is a one-time dose, meaning that the treatment cannot be interrupted. There is no specific experimental protocol to follow after this injection. Instead, the usual medical practices for administering an epidural injection of lidocaine for emergency caesarean section will be followed, and data will be collected.

##### Strategies to improve adherence to interventions {11c}

In order to assure that the correct concentrations and volumes of local anesthetic are administered, the syringes containing either lidocaine with epinephrine (LE) or lidocaine with epinephrine plus bicarbonate (LEB) will be prepared beforehand and stored in a refrigerator near the delivery room for a 24-h period. After the patient’s inclusion in the study, the investigators will have simply to epidurally inject the contents of the prepared syringe that correspond to the group to which the patient has been assigned.

##### Relevant concomitant care permitted or prohibited during the trial {11d}

Before inclusion in the study, the maintenance of epidural analgesia will be ensured by continuous infusion of a mixture of levobupivacaine 1.225% or ropivacaine 1%, combined with sufentanil 0.25 μg/ml delivered through patient-controlled epidural analgesia (PCEA). The management of additional bolus doses of analgesia, if required, will be left to the discretion of the managing anesthesiologist. Obstetrical labor monitoring will be performed in line with current standard of care.

After study inclusion and the injection of the bolus dose of LE or LEB, additional analgesic treatments can be used at the discretion of the managing anesthetist, within the limits of compliance with the main evaluation criterion: the conversion to general anesthesia in the event of pain evaluated at more than 3/10 on the visual numerical scale.

### Provisions for post-trial care {30}

The sponsor and the investigator(s) undertake that this research will be carried out in accordance with the law n°2012-300 of March 5, 2012, relating to research involving the human person, as well as in accordance with the Good Clinical Practices and the Declaration of Helsinki (which can be found in its complete version on the site http://www.wma.net).

The Bordeaux University Hospital, promoter of this research, has taken out a civil liability insurance policy with LLOYD’S INSURANCE COMPANY SA, through the brokerage firm BEAH SAS, in accordance with the public health code.

### Outcomes {12}

Primary outcome:

- Rate of conversion to maternal general anesthesia for insufficient analgesia (defined as the intravenous injection of general anesthetic agents and the need for orotracheal intubation) between the time of the injection of a bolus dose of LE or LEB and clamping of the umbilical cord.

Secondary outcomes:

- Time from the decision to extract the fetus by emergency caesarean section (caesarean decision) to the birth of the baby (the number of minutes from caesarean decision to the clamping of the umbilical cord).
- Time from caesarean decision to incision (the number of minutes from caesarean decision to surgical incision)
- Incidence of maternal complications after the injection of an anesthetic dose of LE or LEB, and in particular that of intraoperative and postoperative nausea or vomiting, arterial oxygen desaturation (SpO2 < 94%), difficult intubation (> 2 direct laryngoscopies), aspiration of gastric contents and/or aspiration pneumonitis, arterial hypotension prior to fetal extraction (MAP < 65 mmHg), extensive sensory or motor block (level ≥ C8)
- Use of adjunct medications necessary for maternal comfort during caesarean section
- Postpartum hemorrhage (bleeding > 500 mL)
- Pediatric wellness criteria (cord pH < 7; cord lactate levels)
- Level of anesthesia assessed 1 h after surgical incision
- Maternal satisfaction with analgesia and anesthesia during caesarean section.

### Participant timeline {13}

**Fig. 1:**
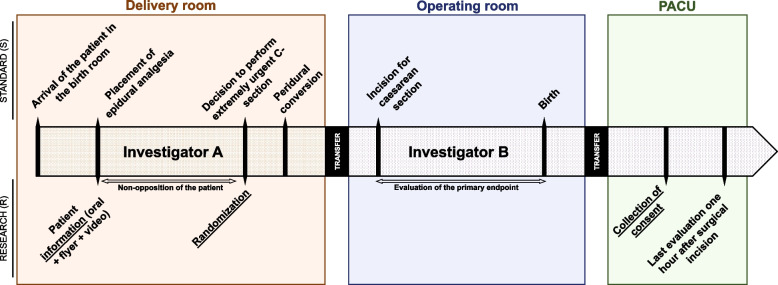
Patients will be informed about the study before receiving epidural analgesia and randomized to one of two treatment arms if emergent fetal extraction is required. The anesthesiologist administering the epidural conversion will be considered “Investigator A,” and the quality of surgical anesthesia will be evaluated by a second anesthesiologist (“Investigator B”). The patient’s written consent will be obtained in the recovery room after the procedure

Patients will receive written information about the QETAL study during the preoperative anesthesia consultation. Upon admission to the delivery room, the anesthesiologist will review the study information again with the patient (see Fig. 1 and Table 2). Epidural analgesia will then be administered according to departmental protocols. Once the decision is made that emergent fetal extraction (>15 min) will be required, the patient will be randomized to either the LE or LEB treatment arm. Because it is impossible to equalize the volume of the injected solution into the epidural space between groups without affecting the level of anesthesia obtained, the control group will receive two 10-mL syringes, totaling 20 mL, while the experimental group will receive a single 12-mL syringe. As a result, it is not possible to use concealed syringes in a way that would allow the administering physician to be unaware of the solution they are injecting or the group to which the patient has been randomized. Consequently, the involvement of two anesthesiologists is necessary. The anesthesiologist who performs the epidural conversion will be designated as “Investigator A” and may delegate this injection to an authorized person. The patient will then be transferred to the operating room for the caesarean section, where the surgical anesthesia quality will be evaluated by the operating obstetrician in the presence of a second anesthesiologist (“Investigator B”). At no point will "Investigator A" communicate the nature or quantity of the solution used for the epidural injection to "Investigator B". Finally, the patient's written consent will be obtained in the recovery room after the procedure.

**Table 2:**
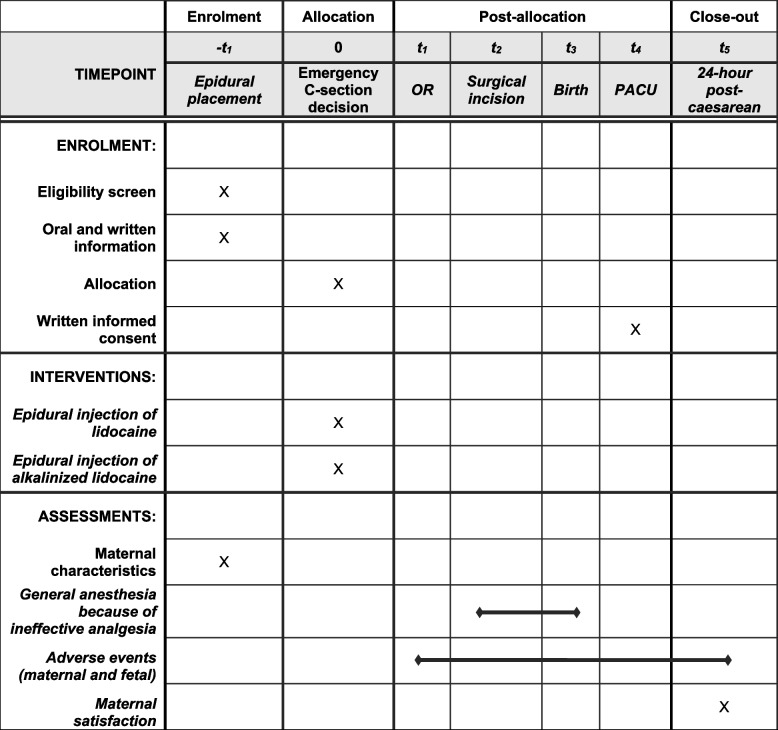
Timing of participant recruitment, administration of interventions, and collection of data

*OR* Operating room, *PACU* Post-anesthesia care unit

### Sample size {14}

No previous study has investigated the use of general anesthesia after epidural extension with an alkalinized local anesthetic solution in extremely emergency caesarean sections during labor. However, a preliminary retrospective clinical audit conducted in 2019 at the Bordeaux University Hospital maternity ward found that the rate of recourse to general anesthesia was 100% (4 out of 4) in the lidocaine alone group, versus 0% (0/7) in the lidocaine-bicarbonate group. Although the sample size was small, the difference was significant (*p*=0.003). Therefore, caution should be exercised when using this preliminary result to calculate the number of subjects required for the current trial. Conservatively, assuming a general anesthesia conversion rate of 80% in the control group and 40% in the experimental group, 44 patients will be required in the lidocaine-bicarbonate group and 22 patients in the lidocaine group, for a total of 66 patients in a 2:1 ratio. The number of subjects required was calculated for a two-sided comparison test of two proportions with a 90% power and a 5% risk alpha.

### Recruitment {15}

Each center will recruit as many patients meeting the eligibility criteria as needed, up to 66 patients.

## Assignment of interventions: allocation

### Sequence generation {16a}

The randomization list will be established by the statistician of the Clinical Epidemiology Unit of the Bordeaux University Hospital before beginning the study. The 2 treatment groups will be unbalanced with a 2:1 ratio in favor of the experimental group (44 subjects in the LEB group and 22 subjects in the LE group). Randomization will be stratified by the investigating center. A document describing the randomization procedure will be kept confidential in the Clinical Epidemiology Unit.

### Concealment mechanism {16b}

To ensure the concealment of the treatment allocation in this randomized study, a web-based system cannot be used due to the extreme emergency of the anesthetic procedure. Therefore, the randomization process will be conducted in advance during the preparation of the refrigerated syringes. The randomization list will be securely stored in a sealed envelope in the birthing room of the investigating centers, accessible only by the nurse anesthetists who are responsible for syringe preparation. This method will ensure that the treatment allocation remains concealed until the point of injection, preventing any potential bias in the study.

### Implementation {16c}

To ensure efficient and accurate randomization, three syringes will be prepared per 24 h. The syringes will be labeled with patient numbers (patient 1, patient 2, or patient 3) based on their corresponding rank in the randomization list for that period. The solutions in the syringes will be identified by batch numbers, and the information of the patient to whom the syringe is dispensed will be recorded along with the date of injection. The syringes will be stored in a secure refrigerator in the delivery room for a maximum of 24 h. Unused syringes will be discarded after 24 h, and the randomization numbers for the unused syringes will be recycled for the following day’s preparations. This method ensures that the randomization process remains confidential and secure.

## Assignment of interventions: blinding

### Who will be blinded {17a}

Participants in the trial will be blinded to their treatment group. The investigator responsible for converting epidural analgesia to epidural anesthesia (referred to as “Investigator A”) will not be blinded, as they will be aware of the difference in volume between the two groups (20 mL versus 12 mL). However, the second investigator (“Investigator B”) who will take care of the patient in the operating room will be blinded, as will the patient’s obstetrician-gynecologist, who will assess the primary endpoint. Biostatisticians involved in the analysis of the data will not be blinded.

### Procedure for unblinding if needed {17b}

Unblinding will only be considered in the event of a serious adverse event that requires knowledge of the patient’s treatment assignment. Under no other circumstances will unblinding be allowed. If unblinding is deemed necessary, the randomization list will be consulted.

## Data collection and management

### Plans for assessment and collection of outcomes {18a}

The data are collected by the investigators at the time of the patient’s inclusion using a four-page sheet containing all the required information. See Additional file 1: “Data collection form.”

### Plans to promote participant retention and complete follow-up {18b}

Due to the short period of patient participation, which spans from the delivery room to PACU, we do not anticipate any patient loss during follow-up. Furthermore, as the only difference between the experimental and control groups is the solution injected during epidural conversion, no specific procedures or outcomes will be collected for patients who deviate from the protocol.

### Data management {19}

The software used for data management is REDCap. It is coupled with a dedicated MySQL database. The Information System Department (ISD) of the Bordeaux University Hospital provides hosting and maintenance of the database according to its procedures. The servers (Internet and database) are hosted in the ISD of the Bordeaux University Hospital (Talence, France). A backup is also hosted in Roubaix (France) and a second one is in a remote site located in Gravelines (France).

The management of the access rights to the software is under the responsibility of the REDCap administrator of the Bordeaux University Hospital. Data entry in the eCRF is under the responsibility of the investigator of the center. Any person other than the investigator who enters data into the eCRF must be trained and delegated by the investigator to do so. Only the data managers, the Clinical Epidemiology Unit team, and the auditors have direct access rights to the database. Prescribed treatments and clinical events are coded in the eCRF in order to perform data control and analysis. Data control are scheduled to verify the consistency and completeness of the data entered into the eCRF. The study data validation plan lists the data controls to be implemented. Requests for corrections are managed by the data manager. They are sent to the investigating center via the eCRF. The investigating team makes the necessary corrections to resolve the correction requests. After all correction requests are resolved, the data manager proceeds to the freezing of the database.

### Confidentiality {27}

In accordance with the law, the persons having direct access to the source data will take all the necessary precautions to ensure the confidentiality of the information relating to the research, to the persons involved, and in particular with regard to their identity and to the results obtained. These persons, as well as the investigators themselves, are subject to professional secrecy.

During the research or at its conclusion, the data collected on the persons who lend themselves to it and transmitted to the promoter by the investigators (or any other specialist) will be made anonymous. Under no circumstances should the names of the persons concerned or their addresses appear in clear text. Each participant will be given a confidential identification code, composed of a center number (2 digits), a patient number (2 digits), and a letter code (3 letters).

The sponsor will ensure that each person who participates in the research has given his or her consent for access to individual data concerning him or her and strictly necessary for the quality control of the research.

### Plans for collection, laboratory evaluation, and storage of biological specimens for genetic or molecular analysis in this trial/future use {33}

Not applicable: no biological specimen.

## Statistical methods

### Statistical methods for primary and secondary outcomes {20a}

The primary statistical analysis will compare the proportion of general anesthesia use between groups LEB and LE. The two groups will be compared using a chi-squared test, a corrected chi-squared test, or a Fisher’s exact test depending on the distribution of the theoretical numbers under the assumption of independence. The two-sided 95% confidence interval will be estimated for each group according to the exact binomial distribution.

Secondary outcomes will be compared according to the following principles. Quantitative variables will be compared by Student’s *t* test, Student’s test for unequal variances, or a non-parametric Wilcoxon test depending on the conditions of application of these tests. Linear regression models will be used to adjust for the major confounders. The assumptions of the model (normality and homoscedasticity of the residuals, linearity of the association for the quantitative factors) will be systematically verified. A transformation of the criteria may be envisaged in the event of deviation from the models’ hypotheses.

Qualitative variables will be compared by a chi-squared test, a corrected chi-squared test, or a Fisher’s exact test according to the distribution of the theoretical numbers under the hypothesis of independence. Logistic regression models will be used to adjust for the major confounders. The hypothesis of the models (log-linearity of the association for the quantitative factors) will be systematically tested.

A graphical representation will be associated as much as possible with the analyses

### Interim analyses {21b}

No interim analysis is planned.

### Methods for additional analyses (e.g., subgroup analyses) {20b}

Not applicable

### Methods in analysis to handle protocol non-adherence and any statistical methods to handle missing data {20c}

Very few missing data are expected in the trial; however, in this case, any missing value of the primary endpoint will be replaced by the value reflecting failure. A sensitivity analysis to missing data will be performed using the maximum bias strategy.

In order to estimate some delays between two partially missing dates, if the day is missing, the day used will be the 15th (middle of the month). If both day and month are missing, value 1 for the day and value 7 for the month (middle of the year) will be used.

### Plans to give access to the full protocol, participant-level data, and statistical code {31c}

Such information may be made available upon specific request to the sponsor.

## Oversight and monitoring

### Composition of the coordinating center and trial steering committee {5d}

The trial steering committee, or “scientific board,” is composed of the principal investigators of the study, a methodologist, biostatisticians, a safety and pharmacovigilance manager, and a representative of the sponsor. This committee meets as required by the study. Its mission is to make any important decision at the request of the coordinating investigator concerning the proper conduct of the research and compliance with the protocol. It verifies compliance with ethics and keeps the Research Department informed of the progress of the research, any problems, and the results available. It decides on any relevant modification of the protocol necessary for the continuation of the research, in particular, measures to facilitate recruitment into the research, modifications of the research documents (protocol, informational and consent forms), decisions to open or close sites participating in the research, measures to ensure the best safety for the persons participating in the research, and discussion of the results and the strategy for the publication of the results. The scientific board may propose to extend or interrupt the research if the rate of inclusion is too slow, if too many patients are lost to follow-up, if there are major violations of the protocol, or for medical and/or administrative reasons. It specifies the possible modalities of prolonged follow-up of the participants included in the research. At the end of each meeting, the president of the scientific board must inform the sponsor of the decisions made. Decisions concerning a major modification or a budget modification must be approved by the sponsor.

### Composition of the data monitoring committee, its role, and reporting structure {21a}

An independent monitoring committee will not be necessary in this study because the treatment received by patients in the “experimental” group (i.e., alkalinized adrenalized lidocaine) will also, in our institution, be received by patients not enrolled in the study since this is also a part of our practice. Patients in the “control” group will only be exposed to treatment that is currently considered to be the gold standard.

### Adverse event reporting and harms {22}

The investigator is responsible for collecting all data related to adverse events that occur between the date of signing the consent form and the end of the participant’s participation. As part of this protocol, all adverse events must be collected. The investigator will report adverse events (clinical and biological) in the observation booklet. These adverse events will be sought at each visit during the study during the interview and clinical examination of the participant. The investigator must notify the Safety and Vigilance Unit by fax or email without delay from the day he/she becomes aware of any adverse event or any new fact related to any adverse event.

### Frequency and plans for auditing trial conduct {23}

An audit may be carried out at any time by persons mandated by the sponsor and independent of those conducting the research. The objective is to verify the safety of the participants and the respect of their rights, the respect of the applicable regulations, and the reliability of the data. An inspection may also be carried out by any competent authority (i.e., ANSM in France).

The audit, as well as the inspection, may apply to all stages of the research, from the development of the protocol to the publication of the results and the classification of the data used or produced in the framework of the research. Investigators agree to comply with the requirements of the sponsor for an audit and the competent authority for an inspection of the research.

### Plans for communicating important protocol amendments to relevant parties (e.g., trial participants, ethical committees) {25}

Any substantial modification, i.e., any modification likely to have a significant impact on the protection of persons, on the conditions of validity, and the results of the research, on the quality and safety of the products tested, on the interpretation of the scientific documents that support the conduct of the research, or on the methods of conducting the research, is the subject of a written amendment that must be submitted to the sponsor; the sponsor must obtain authorization from all regulatory agencies concerned prior to its implementation.

### Dissemination plans {31a}

The analysis of the data provided by the investigating centers is carried out by the USMR of the Bordeaux University Hospital. This analysis will lead to a written report that will be submitted to the sponsor, who will forward it to competent authorities.

Any written or oral communication of the results of the research must receive prior approval from the principal investigator and, if applicable, from any committee set up for the research. The principal investigator undertakes to make available to the public the results of the research, both negative and inconclusive and positive.

The publication of the main results will mention the name of the sponsor; of all the investigators who enrolled or followed participants in the research; of the methodologists, biostatisticians, and data managers who participated in the research; and of the members of the committee(s) set up for the research and the possible participation of the laboratory. The international rules of writing and publication (The Uniform Requirements for Manuscripts of the ICMJE, April 2010) will be taken into account.

In accordance with the law n°2002-303 of March 4, 2002, all participants will be informed, at their request, of the overall results of the research.

## Discussion

Anticipating a significant difference in favor of the lidocaine-bicarbonate group (group LEB), we deemed it ethical to include more patients in that group. As a result, randomization will be unbalanced with a 2:1 ratio, meaning that twice as many patients will be randomized to the experimental group.

Randomization is performed in a context of extreme urgency, within a time frame during which the entire healthcare team is fully mobilized in preparation for the caesarean section.

Although online randomization on a website is fast, the extreme urgency in which the study takes place prohibits its use. Therefore, we needed to set up early randomization. Three treatments, lidocaine alone or lidocaine-bicarbonate, will be prepared and labeled in a randomly defined order every 24 h. The use of a 24-h period is based on product stability. Eligibility criteria in the trial will be scrupulously checked before using the labeled products to limit exclusions after randomization as much as possible. To achieve this objective, the number of inclusion and exclusion criteria has been voluntarily limited.

Another element that will be studied in the analysis of baseline population characteristics will be to compare thermal sensory levels before injection in the two groups to overcome a significant confounding bias. Indeed, beyond the extension solution used, the pre-existing sensory level of epidural analgesia is a determining factor in the speed and extent of epidural extension and ultimately its success or failure. The randomization process is expected to offer a similar distribution of sensory levels before injection, but it will be necessary to ensure that there is no imbalance.

Alkalinization of local anesthetics could improve the success rate of epidural extension by reducing the onset time of surgical anesthesia and therefore the rate of recourse to general anesthesia.

## Trial status

Protocol version number and date: version 1.2 (March 15, 2022)

Date recruitment began: July 7, 2022

When recruitment will be completed: Q3 2023 or Q4 2023

## Supplementary Information

**Additional file 1.** Datacollection form.

**Additional file 2.** Consent form.

**Additional file 3.** Informational private YouTube video.

**Additional file 4.** Patient information sheet.

## Data Availability

Only the database managers, the team in charge of clinical research at the Bordeaux University Hospital and the auditors will have direct access to the database.

## Abbreviations

PACU: Post-Anesthesia Care Unit
SFAR: Société Française d’Anesthésie-Réanimation – French Society of Anaesthesia and Critical Care
CNGOF: Collège National des Gynécologues et Obstétriciens Français - French national college of obstetricians and gynecologists
USMR: Unité de Soutien Méthodologique à la Recherche - Research Methodology Support Unit
ANSM: Agence nationale de sécurité du médicament - French Agency for the Safety of Health Products
