# Supplementary material for: Quick Epidural Top-up with Alkalinized Lidocaine for emergent caesarean delivery (QETAL study): protocol for a randomized, controlled, bicentric trial
Source: Trials. 2023 May 19;24:341. doi: 10.1186/s13063-023-07366-1 (PMC10197428; doi:10.1186/s13063-023-07366-1)
Supplement: Supplementary file 1 — Additional file 1. Datacollection form. [file 13063_2023_7366_MOESM1_ESM.pptx]

## Slide 1
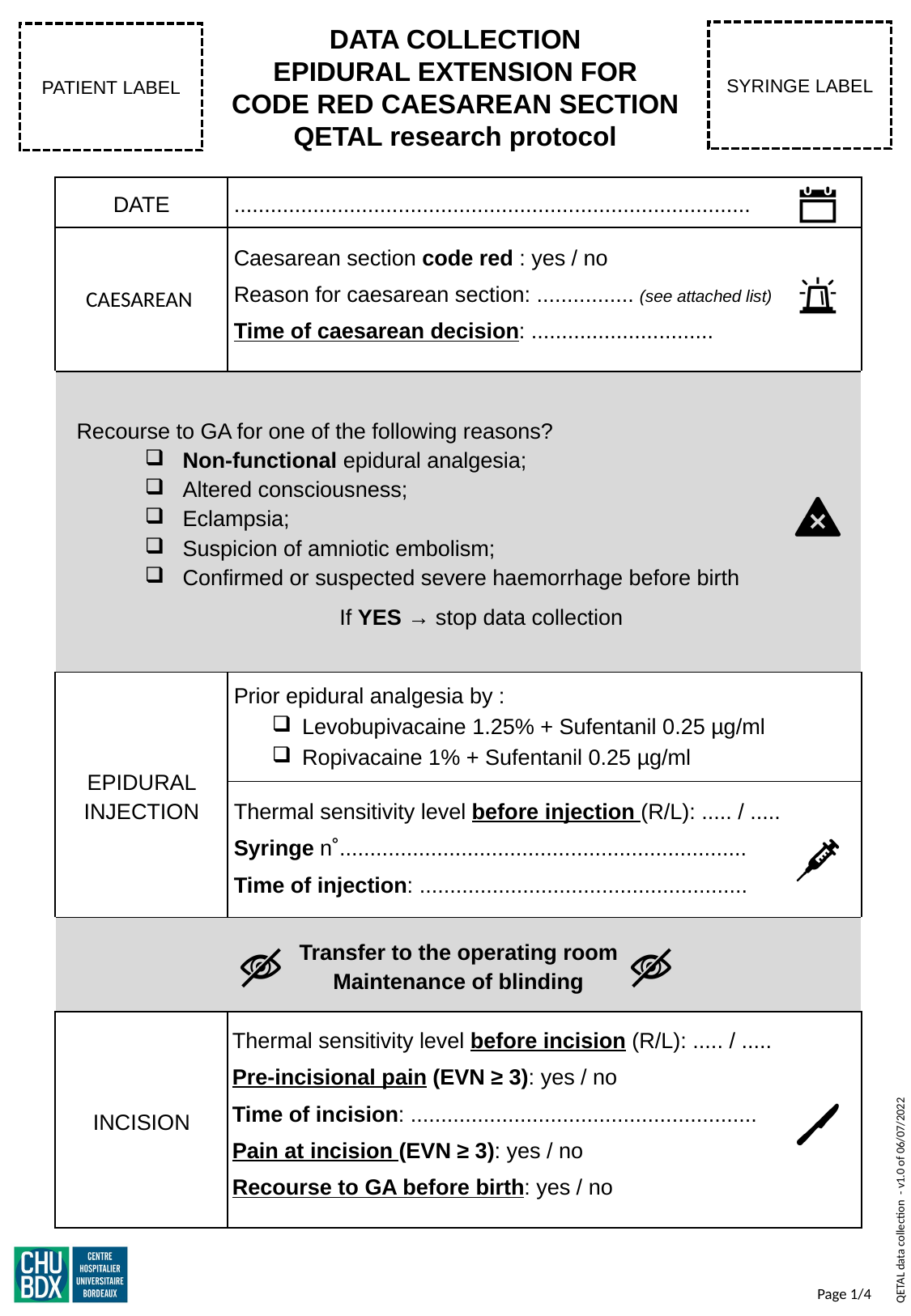

DATA COLLECTION
EPIDURAL EXTENSION FOR
CODE RED CAESAREAN SECTION
QETAL research protocol
SYRINGE LABEL
PATIENT LABEL
| DATE | ..................................................................................... |
| --- | --- |
| CAESAREAN | Caesarean section code red : yes / no Reason for caesarean section: ................ (see attached list) Time of caesarean decision: .............................. |
| Recourse to GA for one of the following reasons? Non-functional epidural analgesia; Altered consciousness; Eclampsia; Suspicion of amniotic embolism; Confirmed or suspected severe haemorrhage before birth If YES → stop data collection | |
| EPIDURAL INJECTION | Prior epidural analgesia by : Levobupivacaine 1.25% + Sufentanil 0.25 µg/ml Ropivacaine 1% + Sufentanil 0.25 µg/ml |
| | Thermal sensitivity level before injection (R/L): ..... / ..... Syringe n˚................................................................... Time of injection: ...................................................... |
| Transfer to the operating room  Maintenance of blinding | |
| INCISION | Thermal sensitivity level before incision (R/L): ..... / ..... Pre-incisional pain (EVN ≥ 3): yes / no Time of incision: ......................................................... Pain at incision (EVN ≥ 3): yes / no Recourse to GA before birth: yes / no |
QETAL data collection - v1.0 of 06/07/2022
Page 1/4

## Slide 2
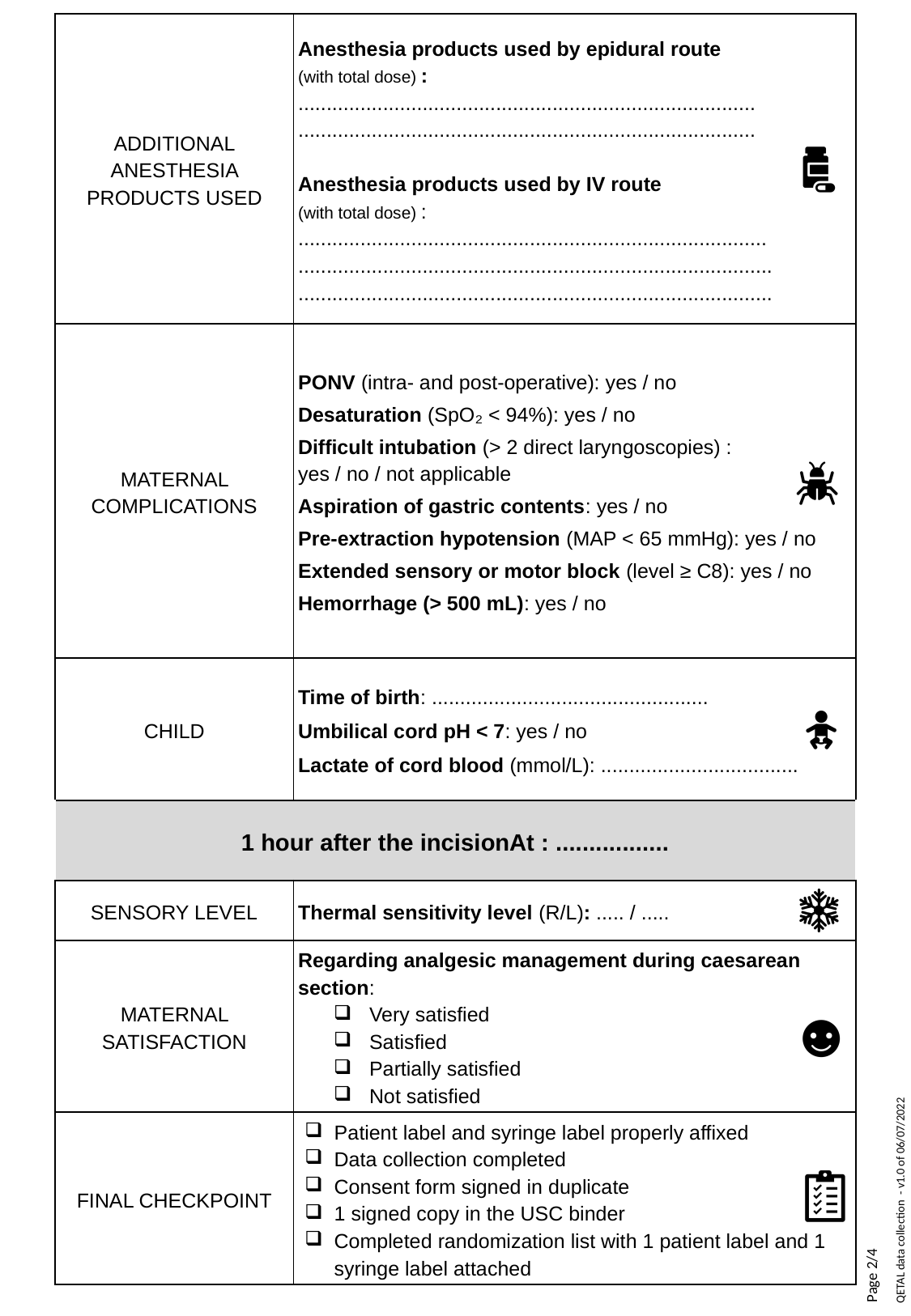

| ADDITIONAL ANESTHESIA PRODUCTS USED | Anesthesia products used by epidural route (with total dose) : ................................................................................. ................................................................................. Anesthesia products used by IV route (with total dose) : ................................................................................... .................................................................................... .................................................................................... |
| --- | --- |
| MATERNAL COMPLICATIONS | PONV (intra- and post-operative): yes / no Desaturation (SpO₂ < 94%): yes / no Difficult intubation (> 2 direct laryngoscopies) : yes / no / not applicable Aspiration of gastric contents: yes / no Pre-extraction hypotension (MAP < 65 mmHg): yes / no Extended sensory or motor block (level ≥ C8): yes / no Hemorrhage (> 500 mL): yes / no |
| CHILD | Time of birth: ................................................. Umbilical cord pH < 7: yes / no Lactate of cord blood (mmol/L): ................................... |
| 1 hour after the incision At : ................. | |
| SENSORY LEVEL | Thermal sensitivity level (R/L): ..... / ..... |
| MATERNAL SATISFACTION | Regarding analgesic management during caesarean section: Very satisfied Satisfied Partially satisfied Not satisfied |
| FINAL CHECKPOINT | Patient label and syringe label properly affixed Data collection completed Consent form signed in duplicate 1 signed copy in the USC binder Completed randomization list with 1 patient label and 1 syringe label attached |
QETAL data collection - v1.0 of 06/07/2022
Page 2/4

## Slide 3
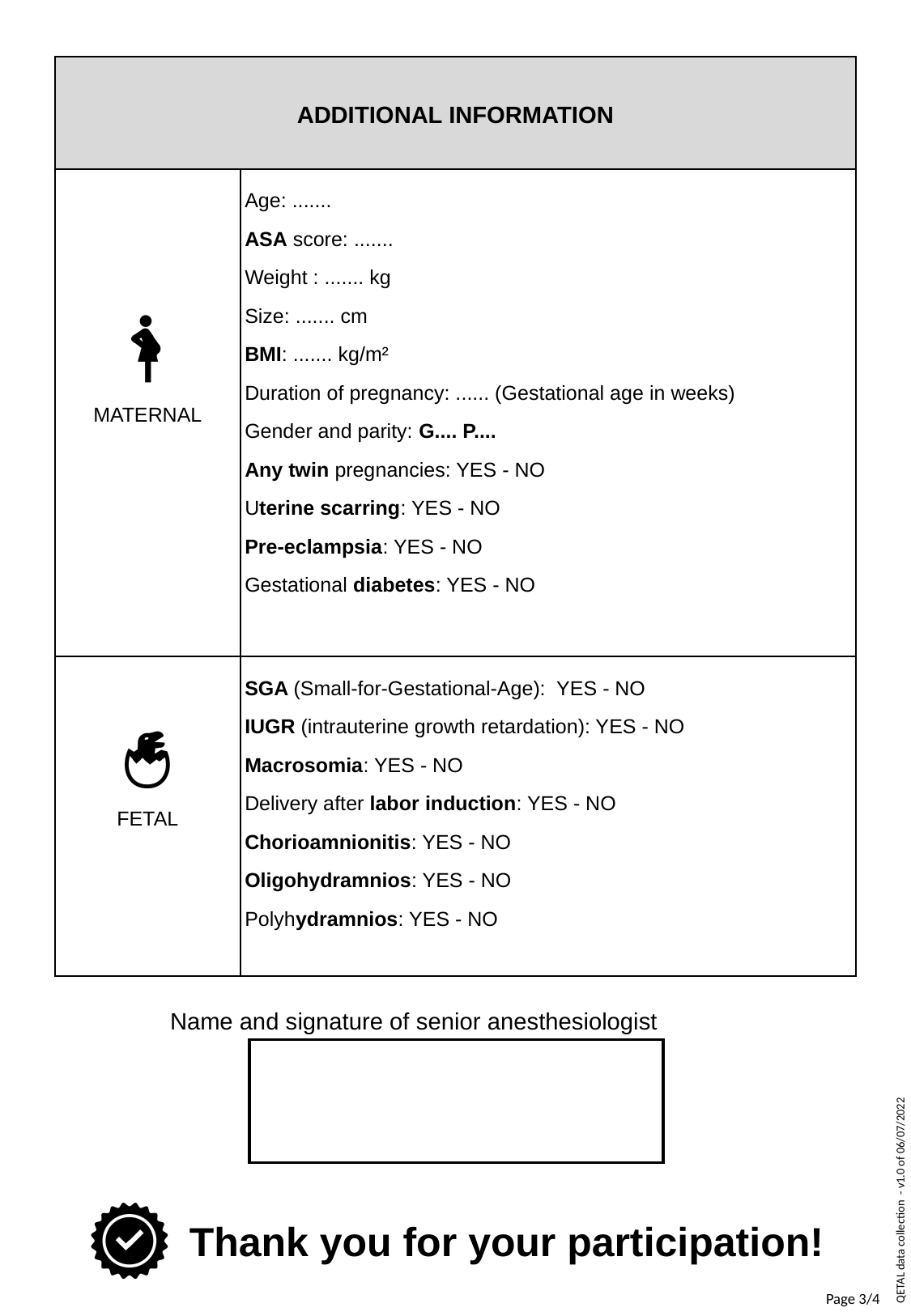

| ADDITIONAL INFORMATION | |
| --- | --- |
| MATERNAL | Age: ....... ASA score: ....... Weight : ....... kg Size: ....... cm BMI: ....... kg/m² Duration of pregnancy: ...... (Gestational age in weeks) Gender and parity: G.... P.... Any twin pregnancies: YES - NO Uterine scarring: YES - NO Pre-eclampsia: YES - NO Gestational diabetes: YES - NO |
| FETAL | SGA (Small-for-Gestational-Age): YES - NO IUGR (intrauterine growth retardation): YES - NO Macrosomia: YES - NO Delivery after labor induction: YES - NO Chorioamnionitis: YES - NO Oligohydramnios: YES - NO Polyhydramnios: YES - NO |
Name and signature of senior anesthesiologist
QETAL data collection - v1.0 of 06/07/2022
Thank you for your participation!
Page 3/4

## Slide 4
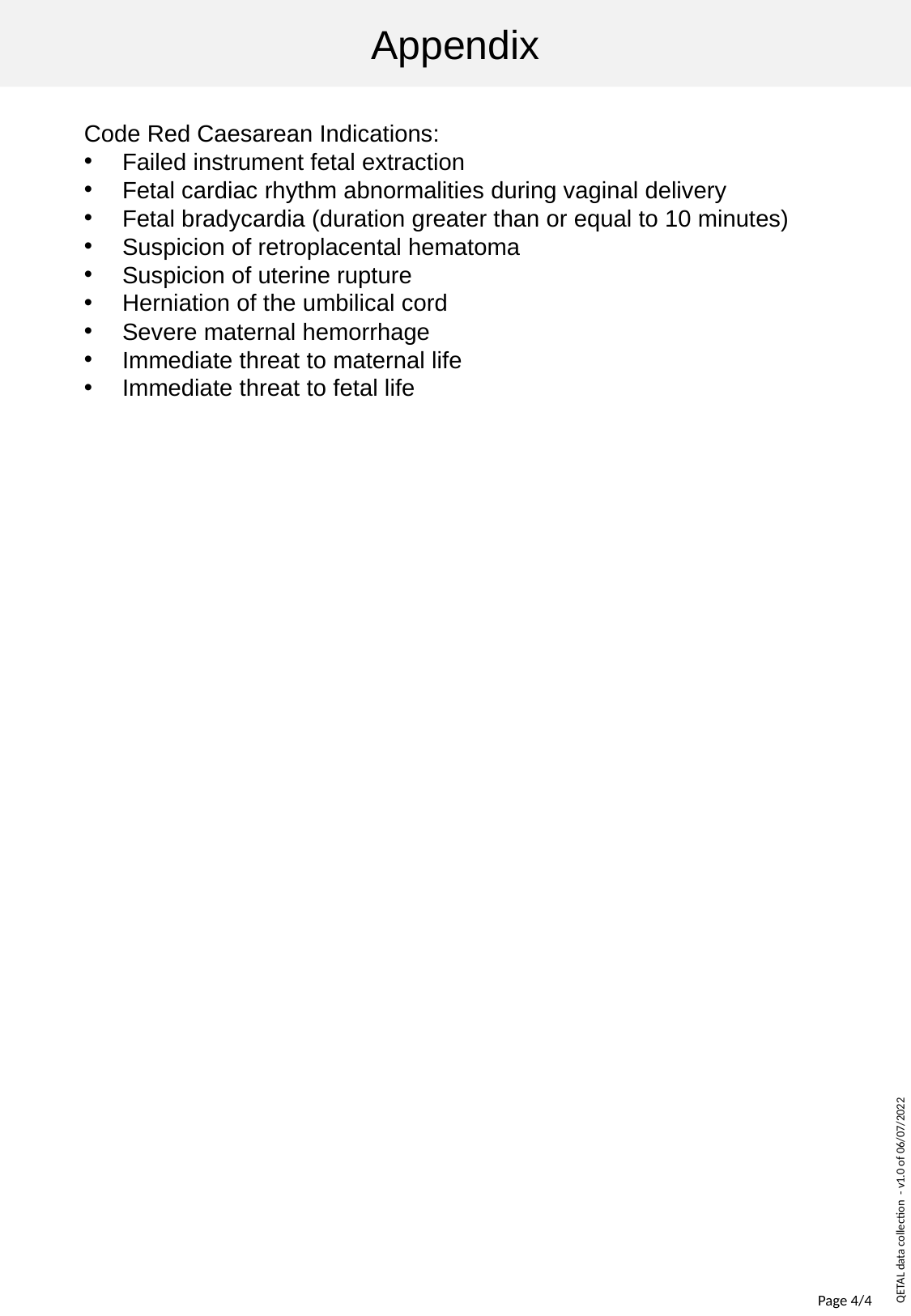

Appendix
Code Red Caesarean Indications:
Failed instrument fetal extraction
Fetal cardiac rhythm abnormalities during vaginal delivery
Fetal bradycardia (duration greater than or equal to 10 minutes)
Suspicion of retroplacental hematoma
Suspicion of uterine rupture
Herniation of the umbilical cord
Severe maternal hemorrhage
Immediate threat to maternal life
Immediate threat to fetal life
QETAL data collection - v1.0 of 06/07/2022
Page 4/4
