# Supplementary material for: Quick Epidural Top-up with Alkalinized Lidocaine for emergent caesarean delivery (QETAL study): protocol for a randomized, controlled, bicentric trial
Source: Trials. 2023 May 19;24:341. doi: 10.1186/s13063-023-07366-1 (PMC10197428; doi:10.1186/s13063-023-07366-1)
Supplement: Supplementary file 2 — Additional file 2. Consent form. [file 13063_2023_7366_MOESM2_ESM.docx]

**Alkalinization of adrenalinized lidocaine in extending epidural analgesia for emergent caesarean section during labor: A randomized controlled trial.**

**QETAL - CHUBX 2021/36**

Sponsor of the research: **CHU de Bordeaux**

Coordinating Investigator: **Dr. Thomas LECHAT**

Madam,

Due to the medical emergency encountered, we were unable to obtain your consent prior to your inclusion and participation in this research, as presented to you by an investigator and by the information given during the epidural procedure.

According to the law (art L1122-1-3 of the public health code) this inclusion without consent is possible in this type of emergency situation.

Nevertheless, now that the conditions are met to express your wishes, your agreement is sought for your participation in this research. Your participation remains entirely voluntary. If you do not wish to take part in this research, you will continue to receive the best possible medical care, according to current knowledge.

**Why this research?**

You have given birth in our maternity hospital, for which you benefited from an epidural catheter by the anesthesia team. The epidural relieved the pain of contractions. The epidural also allowed, in case of emergency, to perform a caesarean section.

Each year, approximately 1 to 2 percent of births require an emergency caesarean section (called "code red" cesarean section in France). When an emergency caesarean section is decided by the obstetrician, additional anesthesia is required to ensure that the surgical procedure is painless. Therefore the anesthesia team injects a powerful local anesthetic into the epidural. This converts the partial anesthesia of the lower body into full anesthesia.

This technique, called "epidural extension" or “epidural top-up” is performed, with a few exceptions, in all emergency caesarean sections if a functional epidural catheter is in place.

However, there is a risk of failure of this technique. In this case, the anesthesia is not effective enough to perform the caesarean section in the short time required by the emergency situation. The anesthesiologists then proceed to a general anesthesia.

Currently, within the medical community, there is a debate about which anesthetic product would be the most effective to perform the epidural extension. At the University Hospital of Bordeaux and the Hospital Center of Bayonne, anesthesiologists use mostly a solution of lidocaine alkalinized with sodium bicarbonate. In the majority of other maternity hospitals in France, a solution of lidocaine alone (without sodium bicarbonate) is used.

To date, no advantage of either solution over the other has been demonstrated. Similarly, no excess risk has been proven with either technique.

This research therefore seeks to verify that reinforcing the epidural with lidocaine solution with sodium bicarbonate is more effective and would therefore reduce the need for general anesthesia in the context of emergent caesarean sections.

**What is the objective of this research?**

The objective of the study is to compare two types of epidural analgesia reinforcement on their effectiveness with respect to the number of times the recourse to general anesthesia in emergent caesarean sections ("code red caesarean ») is required.

**How will this research be conducted?**

When the need for emergency caesarean section is decided by the obstetrician, and if you did not object to participating in this research during the information given earlier, inclusion in the research is carried out and the type of anesthesia will be decided randomly. Neither you nor the doctors can choose the treatment administered. This process, called "randomization", is used in clinical trials to ensure that the groups of people who receive each of the treatments being studied have the same characteristics.

The draw will randomly allocate one of the following epidural reinforcement solutions:

- 20 ml of lidocaine 2% adrenalinized at 0.0005%.
- 10 ml of lidocaine 2% adrenalinized at 0.0005% associated with 2 ml of sodium bicarbonate 4.2%.

Lidocaine is the most commonly used local anesthetic. It is this medication that emergency physicians use to perform sutures and dentists use to numb teeth. Like all local anesthetics, it temporarily interrupts the transmission of impulses in the nerves and thus blocks pain. Lidocaine is the reference local anesthetic for the reinforcement of epidural anesthesia.

Sodium bicarbonate is naturally present in the blood in significant quantities to control blood acidity. We commonly use sodium bicarbonate to reduce blood acidity or the acidity of a product to be injected. We believe that reducing the acidity of lidocaine before injecting it will allow it to be effective more quickly.

The study will be blinded, which means that neither you nor the medical team in the operating room will know the nature of the product assigned.

The duration of your participation is limited to the period between the reinforcement of the epidural for the caesarean section and the period of monitoring in the recovery room after the procedure (about one to two hours). Any adverse events will also be collected during the 24 hours following the caesarean section.

The rest of the medical care is unchanged.

This study is being conducted at 2 institutions: the Bordeaux University Hospital and the Hospital Center of Bayonne, and it is planned to include 66 participants (22 in the lidocaine arm and 44 in the lidocaine plus sodium bicarbonate arm).

**Who can participate?**

To participate in this study you must be of age and affiliated with the social security system in France, have no contraindication to the use of lidocaine or sodium bicarbonate and need an emergency "code red" caesarean section.

**What will you be asked?**

You will be asked, only at the end of the research, your satisfaction with the intervention.

**What are the expected benefits?**

Participation in this study will contribute to the improvement of biological and medical knowledge and in particular will allow the identification of a new epidural reinforcement solution. It is expected that the alkalinized solution will reduce the need for general anesthesia.

**What are the possible disadvantages?**

Your participation in this research does not change the way you would be managed for a caesarean section.

The foreseeable risks are those associated with enhanced epidural analgesia, general anesthesia, and conventional emergency caesarean section surgery during labor.

There is no additional risk to you or your child in connection with the research protocol as defined. In fact, preliminary studies show us that the alkalinization process we are studying is at least as effective as the reference process.

The medical literature does not describe any adverse event related to the epidural administration of sodium bicarbonate.

**What are the possible medical alternatives?**

You have the right not to agree to participate in this research or to stop at any time and for any reason. In this case, your delivery will be managed according to the standard of care.

**What are your rights?**

The investigator who proposes that you take part in this research must provide you with all the necessary explanations concerning this research.

Privacy

To ensure the confidentiality of your personal information, neither your name nor any other information that would directly identify you will be entered into the case report form or any other record that the study investigator provides to the Sponsor, its authorized representatives, or partners. You will only be identified by a code and your initials. The code is used so that the study investigator can identify you if necessary. This data will be stored on secure computer servers.

Processing of personal data

The study investigator and other authorized study personnel will collect information about you and after you have given your consent. Only information that is strictly necessary for the purpose of the research will be collected. This information will be related to your health and your participation in the study. This information is reported on study-specific files, called observation books, provided by the Sponsor.

The information collected will be as follows:

- Information related to anesthesia: products used, effectiveness of anesthesia, whether or not general anesthesia is used;

- Occurrence or not of perioperative adverse events;

- Fetal well-being information at birth;

- Clinical and demographic data related to the patient, her pregnancy, and her fetus.

Within the framework of the research, a computer will process your personal data and analyze the results of the research with regard to the objectives which were presented to you. The collection and processing of this data will be done for scientific research purposes and is based on the public interest of the missions of the Bordeaux University Hospital.

The Bordeaux University Hospital will be in charge of data processing. Its headquarters is located at 12 rue Dubernat - 33404 Talence Cedex.

Transfer of your personal data

The Sponsor may share personal information with regulatory agencies or its research partners. These persons, companies, and agencies may be located in countries or regions different from those in which you reside, in particular in countries where data protection may be different or less restrictive than in France. In this case, the Promoter will take all appropriate measures to keep your personal data confidential and the protection of your data will be equivalent to that guaranteed in the European Union regardless of the country to which the data is transmitted.

Re-use of your data

Your data may also be stored and used in accordance with applicable laws and regulations for further research exclusively for scientific purposes to improve knowledge in the field of anesthesia. You may object to this secondary use by checking the "no" box in the consent form*.*

Access and retention of your data

Representatives mandated by the Sponsor or the competent authorities may also need access to your medical and study records in order to verify the quality of the data collected in the study. These individuals are subject to the same medical confidentiality laws as other medical professionals.

Furthermore, we inform you that your data will be kept for a maximum of two years after the last scientific publication related to the research. The data will then be archived, with very restricted access, in accordance with current regulations on archiving in clinical research. The total duration of data preservation at the end of the research will not exceed 25 years.

Exercising your rights

You have the following rights to your data collected and generated as part of your participation in the research:

- Right of access to data,
- Right to rectify erroneous data,
- Right to erasure of data in case of unlawful processing,
- Right of portability allowing you to obtain the data that you have provided to the institution,
- Right to limit the processing of data, especially if this is challenged.

You also have the right to object to the processing of your data. From then on, no new personal data concerning you will be collected. Exercising this right prevents any further processing of your data and you will not be able to continue your participation in this research. However, any information that has already been collected will be retained and will continue to be used for the purposes of the research.

You may exercise these rights by making a written request to the study investigator who will forward the request to the sponsor. The sponsor will respond to your requests in accordance with its legal and regulatory obligations.

You can also access all your medical data directly or through the intermediary of the doctor of your choice, in application of the provisions of article L1111-7 of the public health code. This right is exercised with the doctor who is following you in the context of the research and who knows your identity.

If you have any questions about the processing of your personal data or your rights associated with this data, you can contact the Data Protection Officer at Bordeaux University Hospital [(mesdonneespersonnelles@chu-bordeaux.fr](mailto:mesdonneespersonnelles@chu-bordeaux.fr)).

If, despite the measures put in place by the Promoter, you feel that your rights are not being respected, you may file a complaint with the supervisory authority for data protection, the Commission Nationale de l'Informatique et des Libertés (CNIL). This complaint to the CNIL can be made to the address: <https://www.cnil.fr/fr/webform/adresser-une-plainte>.

Regulatory Considerations:

In accordance with the law n°2012-300 of March 5, 2012 related to human research:

- this research obtained a favorable opinion from the Ile de France III Committee for the Protection of Individuals on 03/14/2022 and authorization from the National Agency for the Safety of Medicines and Health Products (ANSM) on 03/14/2022.

- the Sponsor of this research, the Bordeaux University Hospital (12, rue Dubernat, 33404 Talence cedex), has taken out a civil liability insurance policy (B1339CTLICNWL21-105) with LLOYD'S INSURANCE COMPANY SA through the brokerage firm BEAH SAS, whose address is 16-18 rue de Londres, 75009 Paris (tel.: 04 82 25 01 62)

- the Sponsor assumes responsibility for compensation for any harmful consequences of research involving human subjects.

Search Results:

The results of this research may lead to patented innovations or innovations protected by intellectual property law, and to scientific publications.

When this research is completed, you will be personally informed of the overall results by your doctor as soon as they become available, and if you wish.

After reading this information note, please feel free to ask any questions you may have. If you agree to participate in this research, you must complete and sign the consent form. You will be given a copy of the complete document.

Thank you for your attention.

CONSENT FORM

*Alkalinization of adrenalinized lidocaine in extending epidural analgesia for emergent cesarean section during labor: A randomized controlled trial*

**QETAL - PROMOTER CODE**

Sponsor of the research: **CHU de Bordeaux**

*Coordinating* Investigator: **Dr. Thomas LECHAT**

I, the undersigned *(last name, first name)* certify that I have read and understood the information note that has been given to me.

I had the opportunity to ask all the questions I wanted to the Pr/Dr *(last name, first name)* who explained to me the nature, objectives, potential risks and constraints related to my participation in this research.

I am aware of the possibility of interrupting my participation in this research at any time without having to justify my decision and I will do my best to inform the investigator who is following me in the research. This will, of course, not affect the quality of subsequent care.

I have been assured that the decisions that are necessary for my health will be made at all times, according to the current state of medical knowledge.

I have been informed that this research has received the favorable opinion of the Comité de Protection des Personnes Ile de France III and the authorization of the ANSM (category 1).

The Bordeaux University Hospital has signed a commitment to comply with the "Reference Methodology" (MR-001) in application of the provisions of article 54 paragraph 5 of the modified law of January 6, 1978 relating to information, files, and freedoms.

The Sponsor of the research (CHU de Bordeaux, 12 rue Dubernat, 33404 Talence Cedex) has taken out a civil liability insurance policy in the event of damage with LLOYD'S INSURANCE COMPANY SA through the brokerage firm BEAH SAS, whose address is 16-18 rue de Londres, 75009 Paris (tel: 04 82 25 01 62).

I agree that the persons who collaborate in this research or who are mandated by the Sponsor, as well as possibly the representative of the Health Authorities, have access to the information in the strictest confidentiality.

I agree that the data recorded during this research, in an encoded form, may be subject to computerized processing under the responsibility of the Sponsor. I am informed that, at the end of this research, these data may be used for other research purposes (new statistical analyses) and I have been informed of my right to object to this*.*

I have noted that, in accordance with the General Data Protection Regulation (GDPR) and the provisions of the law relating to data processing, files and freedoms, I have a right of access, rectification, deletion, limitation of processing, portability of data, opposition, and withdrawal. These rights can be exercised with the investigator who is following me in this research and who knows my identity.

My consent does not relieve the Investigator and the Sponsor of the research of their responsibilities to me. I retain all rights guaranteed by law.

The overall results of the research will be communicated to me directly, if I so wish, in accordance with the law of March 4, 2002 on the rights of patients and the quality of the health system.

Having had sufficient time to reflect before making my decision, I freely and voluntarily agree to participate in the QETAL research study

*I freely and voluntarily agree that my clinical data may be stored and used for further research in the field of anesthesia (new statistical analyses)* ❒ *yes* ❒ *no*

**I may at any time request additional information from the investigator who proposed me to participate in this research,
phone number:**

Done at: on: Done at: on :

Signature of participant: Signature of investigator:
