# Supplementary material for: Quick Epidural Top-up with Alkalinized Lidocaine for emergent caesarean delivery (QETAL study): protocol for a randomized, controlled, bicentric trial
Source: Trials. 2023 May 19;24:341. doi: 10.1186/s13063-023-07366-1 (PMC10197428; doi:10.1186/s13063-023-07366-1)
Supplement: Supplementary file 3 — Additional file 3. Informational private YouTube video. [file 13063_2023_7366_MOESM3_ESM.docx]

***Alkalinization of adrenalinized lidocaine in extending epidural analgesia for*
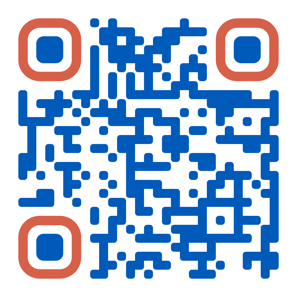
*emergent caesarean section during labor: A randomized controlled trial.***

**QETAL study - Informational video for participants**

<https://youtu.be/JNabPb1vlRk>

Script :

Hello! If you are watching this video, it is because you are getting ready to have your baby in our maternity hospital. Congratulations from all of us!

A partnership of the Hospital of Bayonne and the University Hospital of Bordeaux has formed a collaboration in a clinical research project that may concern you. The purpose of this video is to give you information about this study.

As you probably already know, the epidural is a technique of regional anesthesia. Specifically, it involves a small plastic catheter that is inserted into the epidural space. Through this catheter, we inject a local anesthetic continuously that helps to alleviate the pain in the lower half of the body during labor. The anesthesia is not complete. The goal is that the laboring mother continue to feel her uterine contractions without feeling pain. Thus, the epidural permits a longer labor with much less pain. The sensations of the uterine contractions will remain, which is why we speak in terms of pain relief and not complete anesthesia.

In the event that there is a risk for you or your baby, the obstetrical team may decide to do an emergency caesarean section. If this happens, the anesthesia team will inject a potent anesthetic agent in the epidural catheter. This technique is called the extension or reinforcement of the epidural. This is very effective and in the vast majority of cases allows us to completely anesthetize the lower half of the body for the caesarean without having to use general anesthesia. Unfortunately, in certain cases, the anesthesia by extension of the epidural is incomplete, and we are obligated to have you go completely to sleep to be able to do the caesarean. In this case, the general anesthesia is for a short duration and you will be able to be reunited with your baby immediately upon awakening in the recovery room.

Our study is interested in comparing two techniques of epidural extension. The active medication for epidural extension is the same : lidocaine. This is what is used in the emergency room to anesthetize the skin before placing stitches to close a wound. The formulation of lidocaine can be associated with adrenaline, which is used to augment its effectiveness. The recommendation of the French Society of Anesthesia and Critical Care is to use this formulation (lidocaine plus adrenaline) for an epidural extension. In our centers, we also add sodium bicarbonate. Sodium bicarbonate is present naturally in blood. Its use in epidurals is without risk, and we believe that its use increases the efficacy of the lidocaine. This, however, has not been proved.

Our study, therefore, centers around the technique of epidural extension. To recap, when this technique is successful, we can avoid general anesthesia for emergency caesarean delivery. In case of failure, we would need to perform a general anesthetic of short duration.

In order to look at the efficacy of these two techniques of epidural extension, we can compare their rates of failure by looking at the number of general anesthetics done in each of the two groups. At the present time, we don’t know which will be the most effective. But we do know that neither group will carry more risk than the other.

Violà ! Now you have all the information about our study. Rest assured that our priority, in every instant, is you and your baby.

The anesthesia team is at your disposal for any and all additional information.

Thank you for your attention. And we wish you a wonderful delivery in our maternity unit!
