# Supplementary material for: Quick Epidural Top-up with Alkalinized Lidocaine for emergent caesarean delivery (QETAL study): protocol for a randomized, controlled, bicentric trial
Source: Trials. 2023 May 19;24:341. doi: 10.1186/s13063-023-07366-1 (PMC10197428; doi:10.1186/s13063-023-07366-1)
Supplement: Supplementary file 4 — Additional file 4. Patient information sheet. [file 13063_2023_7366_MOESM4_ESM.docx]

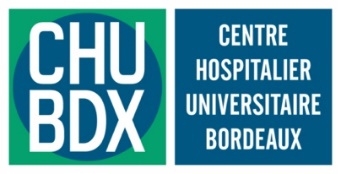
 QETAL - CHUBX 2021/36
Information flyer version 1.1 of 03/15/2022


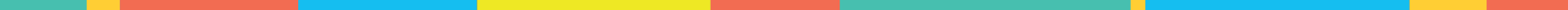


**Alkalinization of adrenalinized lidocaine in extending epidural analgesia for emergent caesarean section during labor: a randomized controlled trial.**

Congratulations! You are about to give birth in our maternity ward. The purpose of this information sheet is to inform you about an ongoing study in our department that may concern you.


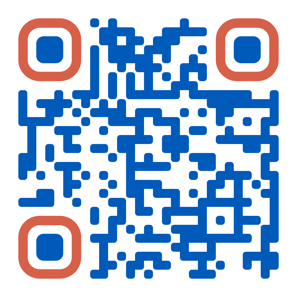
This information is also available in video format:

The entire anesthesia team is at your disposal for any additional information

Preliminary information and consent to participate in the study.

This research cannot take place without your consent. The law requires that your consent be free, informed, and in writing. A consent, signed by you and a doctor, is therefore necessary. However, the conditions under which this research is carried out do not allow, in practice, to collect this signature before your potential inclusion. Thus, if you agree to participate in this research, and only if you are included, you will be asked to sign a consent form at the end of the research, which includes all the information contained in this document and which also details your rights.

You can ask the anesthesia team for a copy of this consent form.

This study has been approved by the committee for the protection of human rights. This committee has ensured that the research is ethical, and that you are protected in your participation in this research.

Why this research?


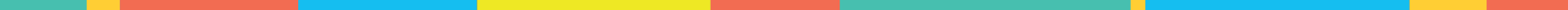


Each year, 5,600 babies are born at the Bordeaux University Hospital, of which 1,200 are born by caesarean section. Some of these caesarean sections are performed without urgency, and others must be performed emergently. When the obstetrician decides to perform an emergency caesarean section, the anesthesia team's mission is to do everything possible to perform this act as quickly as possible, without pain for the patient or risk to the fetus

The anesthesia team then injects a powerful local anesthetic into the existing epidural, which converts the partial lower body anesthesia to full anesthesia. This technique is called an "epidural extension" or "epidural top-up" and is performed, with few exceptions, in all emergency caesarean sections if there is a functional epidural catheter in place.

However, there is a risk of failure of this technique. In this case, the anesthesia is not effective quickly enough to perform the caesarean section in the short time required by the urgency of the situation. The anesthesiologists must then proceed to a general anesthetic before performing the caesarean section (see Figure 1).

Currently, within the medical community, we do not know which anesthetic product is the most effective to perform the epidural extension. At the University Hospital of Bordeaux and at the Hospital Center of Bayonne, the teams of anesthesiologists mostly use a solution of lidocaine alkalinized with sodium bicarbonate. In the majority of other maternity hospitals in France, a solution of lidocaine alone (without sodium bicarbonate) is used.

To date, no advantage of one solution over the other has been demonstrated. Similarly, no excess risk has been proved with either technique.


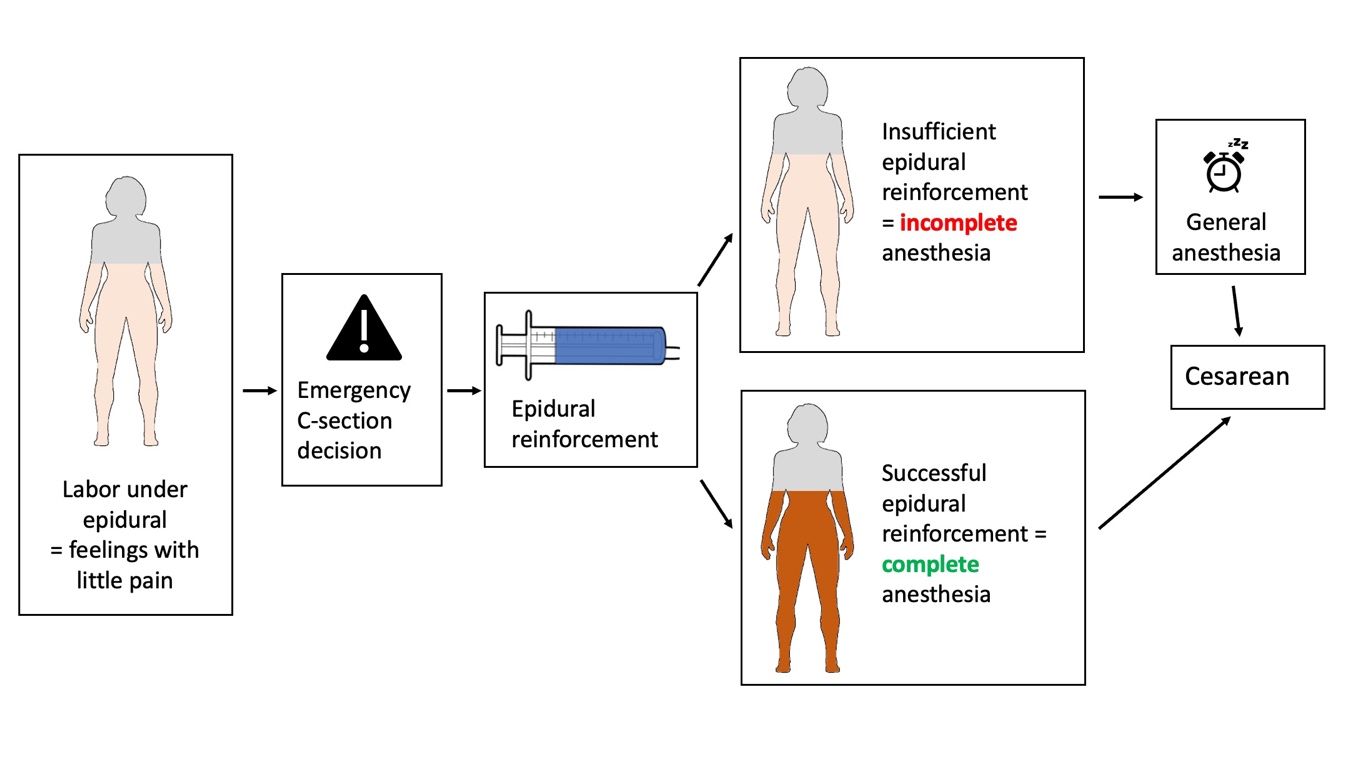


Figure 1 General principle of anesthetic management during emergency caesarean section in labor under previously functional epidural analgesia.

What is the objective of this research?


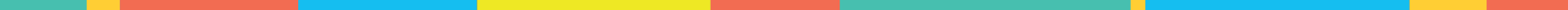


This research seeks to compare two types of epidural analgesia reinforcement with regard to their effectiveness, especially with regard to the number of occasions recourse to general anesthesia is required for emergent cesarean deliveries ("code red cesareans »).

How will this research be conducted?

When the decision to perform an emergency caesarean section is made by the obstetrician, and if you did not object to participating in this research during the information given earlier, inclusion in the research is carried out and the type of anesthesia (lidocaine with adrenaline alone or lidocaine with both adrenaline and bicarbonate) will be decided randomly. Neither you nor the doctors will be able to choose the treatment administered.

The draw will randomly allocate one of the following epidural reinforcement solutions:

- 20 ml of lidocaine 2% adrenalinized at 0.0005%.
- 10 ml of lidocaine 2% adrenalinized at 0.0005% associated with 2 ml of sodium bicarbonate 4.2%.

Lidocaine is the most widely used local anesthetic in the world. You have probably already had it if you have had stitches in the emergency room or during procedures at your dentist's office. Like all local anesthetics, it temporarily interrupts the transmission of nerve impulses and thus blocks the transmission of pain. Lidocaine is also the reference local anesthetic for the reinforcement of epidural anesthesia. Normally, it is combined with low doses of adrenaline to improve its effectiveness.

Sodium bicarbonate is naturally present in the blood in significant quantities to control the acidity of the body. We commonly use sodium bicarbonate to reduce the acidity of blood or of a medication to be injected. We believe that reducing the acidity of lidocaine before it is injected will allow for a more rapid effectiveness of its anesthetic action.

The study will be blinded, which means that neither you nor the medical team in the operating room will know the nature of the product to which you have been assigned.

The duration of your participation will be approximately 24 hours. This will extend from the reinforcement of the epidural for the caesarean section in the birth room, to a 24-hour post-operative follow-up.

The rest of the medical care will be unchanged.

This study is being conducted in 2 institutions: the Bordeaux University Hospital and the Bayonne University Hospital and it is planned to include 66 participants.

Who can participate?


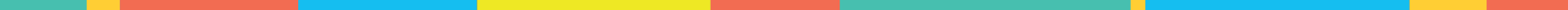


To participate in this study, you must be of legal age and affiliated with the social security system of France, have no contraindication to the use of lidocaine or sodium bicarbonate, and require an emergency "code red" caesarean section.

What will you be asked?

You will be asked, only at the end of the research, your satisfaction with the intervention. The signature of the consent for participation in the research will also be collected at this time.

What are the expected benefits?

Participation in this study will contribute to the improvement of biological and medical knowledge and, in particular, will allow the identification of a new solution for epidural reinforcement. It is expected that this new solution will reduce the need for general anesthesia.

What are the possible disadvantages?

Your participation in this research does not change the way you would be managed for a caesarean delivery.

The foreseeable risks are those associated with enhanced epidural analgesia, general anesthesia, and conventional emergency caesarean section surgery during labor.

There is no additional risk to you or your child in connection with the research protocol as defined. In fact, preliminary studies show us that the alkalinization process we are studying is at least as effective as the reference process.

The medical literature has not identified any adverse event related to the epidural administration of sodium bicarbonate.

What are the possible medical alternatives?

You have the right not to agree to participate in this research or to stop at any time and for any reason. In this case, your care will follow the usual practices of the medical team and may include the injection of an epidural reinforcement solution including sodium bicarbonate, since this is already an accepted form of treatment.
